# Maize Growth Responses to a Humic Product in Iowa Production Fields: An Extensive Approach

**DOI:** 10.3389/fpls.2021.778603

**Published:** 2022-01-10

**Authors:** Daniel C. Olk, Dana L. Dinnes, Chad R. Callaway

**Affiliations:** ^1^National Laboratory for Agriculture and the Environment, U.S. Department of Agriculture—Agricultural Research Service, Ames, IA, United States; ^2^Ag Logic Distributors, Conrad, IA, United States

**Keywords:** humic product, maize, grain yield, leaf area, on-farm survey

## Abstract

Field evaluations of commercial humic products have seldom involved replication across location or year. To evaluate the consistency of humic product efficacy in field conditions, we determined the effects of a humic product on maize (*Zea mays* L.) growth in high-yielding Midwestern (US) fields through the following two extensive approaches: (i) replicated strip plots in five site—year combinations from 2010 to 2013; and (ii) demonstration strips in 30–35 production fields annually from 2009 to 2011 that covered major areas of Iowa. Mechanized combine measurements of grain yield showed increases of 0.2–0.4 Mg ha^–1^ (1–4%) with humic product application for all five site—year combinations of the replicated strip plots. Six of 10 humic treatments within the fields responded positively (*P* < 0.07), and the positive responses of two more treatments approached significance at the benchmark of *P* = 0.10. In the demonstration strips, maize grain weight in hand-collected samples increased significantly (*P* < 0.004) with humic product application in each of the three growing seasons, and across all the three seasons by 6.5% (*P* < 0.001). Grain weight increased numerically for 76 of the 98 demonstration strips. Yield component analysis for both the replicated strip plots and the demonstration strips attributed the yield boosts largely to increased ear length, especially of the shorter ears. Humic product application caused significantly (*P* < 0.10) greater total leaf area in all eight field treatments at three site—year combinations. Humic product application did not consistently affect nutrient concentrations of the grain or stover or any measured soil property. These results represent among the widest geographic evaluations published on field efficacy of a humic product. They demonstrate the capability of a humic product to improve maize growth in high-yielding conditions.

## Introduction

Humic products have received increasing attention as a management tool for increasing crop growth. Plant responses have been demonstrated most often under controlled conditions (Chen and Aviad, 1990; Rose et al., 2014). A growing number of published studies address the field efficacy of humic products for horticultural crops (Bryla and Vargas, 2013; Shahin et al., 2015; Suman et al., 2016; Popescu and Popescu, 2018), agronomic crops (Herrera et al., 2016; Chen et al., 2017; Lenssen et al., 2019; Izquierdo and Pintos, 2021; Pačuta et al., 2021), and in the alleviation of environmental stresses (Osman and Rady, 2012; Bezuglova et al., 2019; Nazli et al., 2020; Qin and Leskovar, 2020; Fallahi et al., 2021; Lindsey et al., 2021). Reviews of field studies were provided by Calvo et al. (2014), Canellas et al. (2015), and Olk et al. (2018). The field studies, however, largely involved only one or two site–year combinations. A notable exception reported significant increases in soybean yield components collected by hand in Uruguayan farm trials, which across 6 years amounted to 85 sites (Izquierdo and Pintos, 2021). Overall, there is a paucity of results on humic product efficacy for numerous field locations and across years. Hence the question of whether positive crop responses to humic products can be generally expected across wide settings in crop production remains unanswered.

To address the above question, this study was conducted in US Midwestern production fields in the maize [*Zea mays* (L.)] phase of a maize---soybean [*Glycine Max* (L.) Merr.] rotation, primarily in central Iowa. Little published evidence exists on humic product efficacy in this region. We measured the maize crop responses to a previous formulation of a liquid humic product, Yield Igniter^®^,^1^ created through alkaline extraction of leonardite ore. Humic product efficacy was evaluated through two complementary approaches. First, we measured maize grain yield through mechanized-combine and yield-component samples for five site—year combinations at three production field trials in central Iowa. These studies had replicated field-long treatment strips which compared humic product applications to unamended controls. In three of these five, we measured leaf area, presuming that the area of each leaf reflects the favorability of growing conditions at the time when the leaf developed (Eik and Hanway, 1965). Second, for a much broader survey of on-farm fields, we determined maize biomass and grain weight in yield-component samples that were hand-collected at physiological maturity from demonstration strips of humic product application, paired with corresponding yield-component samples from adjacent, unamended maize rows. Such paired samples were collected from 30 to 35 production fields annually for three growing seasons (2009–2011), mostly in central Iowa but also including additional sites across Iowa and from Nebraska (NE) and South Dakota (SD). This supplemental approach is intended to determine the trends across a wider geographic region, but limited resource and logistical challenges during its implementation compelled some sacrifices in scientific rigor. The combination of both approaches is intended to provide a uniquely extensive yet replicated database for evaluating the magnitude and reproducibility of maize grain yield responses to this humic product under conventional on-farm production practices in a high-yielding region. This study does not address potential mechanistic explanations for such responses.

## Materials and Methods

### Study Sites

#### Weather Patterns

Field experiments with replicated field-long treatment strips were conducted in 2010, 2011, and 2013 at three sites near the communities of Conrad and Radcliffe in central Iowa. The region is characterized by warm, subhumid summers and cold winters. Maize production in Iowa is rainfed and has traditionally displayed drought stress symptoms in July and August. In this study, annual weather patterns are described locally by measurements collected at the National Oceanic and Atmospheric Administration-National Weather Service weather station site in Marshalltown, about 22 km south of Conrad and 52 km southeast of Radcliffe.

In 2010, the total annual precipitation was 176 mm above the 30-year annual average (1971–2000) (Table 1). Monthly totals during the growing season (April–September) were all above average. Monthly mean temperatures during these same months did not vary dramatically from the 30-year means except for the warm August. In short, growing season conditions were mostly favorable for crop production, aside from the customary summer drought. In 2011, a dry period extended from June through October. Total annual precipitation in 2011 was 179 mm below the average. Both sites experienced the same conditions as most of Iowa: favorable growing conditions in the early part of the growing season, followed by crop drought stress during the second half. In 2013, 566 mm of precipitation fell in April and May, nearly triple the long-term average (192 mm). The remainder of the 2013 growing season, June–October, reverted to drier than normal conditions with a total of 259 mm of precipitation, 277 mm below the average. The wet soil conditions of the early growing season thus abruptly turned to dry conditions beginning in June. The annual mean temperature for 2013 was only 0.9°C below the average.

**TABLE 1:** Monthly precipitation amounts and mean temperatures in 2010, 2011, and 2013, and their deviations from 30-year means (1971–2000), for the replicated field trials in central Iowa.

	Jan.	Feb.	Mar.	Apr.	May	June	July	Aug.	Sep.	Oct.	Nov.	Dec.	Annual
	**Monthly precipitation (mm)**												

30-Yr Mean	24	27	61	84	108	142	116	122	90	67	55	31	927
2010	19	26	15	110	140	201	156	150	204	13	44	20	1103
*Deviation*	*–5*	*–1*	*–46*	*26*	*32*	*59*	*40*	*28*	*114*	*–54*	*–11*	*–11*	*176*
2011	20	10	29	104	130	117	83	31	69	36	58	61	748
*Deviation*	*–4*	*–17*	*-32*	*20*	*22*	*–25*	*–33*	*–91*	*–21*	*–31*	*3*	*30*	*–179*
2013	30	34	59	161	405	100	40	4	55	60	62	20	1029
*Deviation*	*6*	*7*	*–2*	*77*	*297*	*–42*	*–76*	*–118*	*–35*	*–7*	*7*	*–11*	*102*

	**Monthly mean temperature°C**												

30-Yr Mean	–8.2	–4.7	2.1	9.1	15.6	21.0	23.1	21.6	17.0	10.3	2.1	–5.4	8.6
2010	–10.4	–9.8	2.8	12.4	15.5	21.9	2.5	23.7	16.0	11.3	3.0	–7.6	8.5
*Deviation*	*–2.2*	*–5.1*	*0.7*	*3.3*	*–0.2*	*0.9*	*0.5*	*2.2*	*–1.0*	*1.0*	*1.0*	*–2.2*	*–0.1*
2011	–10.0	–5.2	0.8	7.7	14.8	20.7	25.4	21.8	15.1	11.5	4.1	–1.6	8.8
*Deviation*	*–1.8*	*–0.5*	*–1.3*	*–1.4*	*–0.8*	*–0.3*	*2.3*	*0.2*	*–1.9*	*1.2*	*2.0*	*3.8*	*0.1*
2013	–6.3	–5.4	–2.8	6.1	15.0	20.7	22.1	22.1	19.2	10.2	0.4	*-*9.0	7.7
*Deviation*	*2.0*	*–0.7*	*–4.9*	*–3.0*	*–0.7*	*–0.3*	*–1.0*	*0.5*	*2.2*	*–0.1*	*–1.7*	*–3.6*	*–0.9*

The on-farm survey was conducted in 2009–2011, for which we describe state-averaged weather patterns in Iowa. Temperatures in 2009 were mostly cool (Iowa Department of Agriculture and Land Stewardship, 2009), especially in the midsummer months. State annual precipitation averaged 1,017 mm, 10% above the long-term average of 927 mm. This combination of little heat stress and moderate precipitation, particularly during the growing season, made 2009 a favorable year for crop production.

In 2010, the state annual precipitation was 1,146 mm statewide, 24% above the long-term average and the second wettest year in the 138-year record of the state at that time. Every month, except October, had greater than average precipitation and the year began with a heavy amount of snowpack that served to saturate the soil profiles in the early growing season. Temperatures in 2010 during the summer months were marginally warmer than the average, except for the month of August when the monthly mean temperature was 2.2°C greater than the 30-year mean. For 2011, temperatures were moderate to slightly cooler than normal for January through June. That trend was broken in July with the temperature above the normal, and episodic high temperatures over 38°C at some locations in the state in August. Precipitation across the state varied widely but was generally dry, similar to the Marshalltown weather station. By November, 68% of Iowa was classified as being in a drought condition. Hence, conditions were mostly favorable for the first half of the growing season, followed by soil moisture deficits in the second half.

#### Soil Types

##### Central Iowa Trials With Replicated Treatment Strips

Soils in central Iowa were formed on recent glacial till of the Des Moines Lobe (Wisconsin glaciation period), with a cover of wind-blown loess, and are highly productive for crop production. Treatment strips in a study near Radcliffe, IA, traversed all three Mollisols of the Clarion (fine-loamy, mixed, mesic Typic Hapludoll)-Nicollet (fine-loamy, mixed, mesic Aquic Hapludoll)-Webster (fine-loamy, mixed, mesic Typic Haplaquoll) soil association (USDA Soil Conservation Service, 1985b). All three soils have deep and fertile surface soil horizons, with high soil organic matter and good water-holding capacity. For example, the 2010 soil sampling found a mean soil organic matter content (loss on ignition) of 37.9 g kg^–1^, pH (1:1 water) of 6.42, and cation exchange capacity (sum of NH_4_-extractable cations) of 17.4 cmol_*c*_ kg^–1^.

Two replicated studies were also conducted within 2 km of each other near Conrad, IA. Both fields were mapped within the Tama-Muscatine-Downs soil association (USDA Soil Conservation Service, 1977), which are Mollisols with deep surface horizons of high fertility, soil organic matter content, and water-holding capacity. The field on the Ag Logic Distributors research farm (“Conrad” field) consisted predominantly of the Tama soil (fine-silty, mixed, mesic Typic Argiudoll), with a small inclusion of Sawmill silt loam soil (fine-silty, mixed, mesic Cumulic Haplaquoll) in a natural drainage path. Treatment strips at the nearby on-farm “Whitten” field included the Tama, Muscatine (fine-silty, mixed, mesic Aquic Hapludoll), Garwin (fine-silty, mixed, mesic Typic Haplaquoll), and Sawmill soil types. A 2010 soil sampling in this field reported a mean soil organic matter content of 52.1 g kg^–1^, pH of 6.54, and cation exchange capacity of 23.6 cmol_*c*_ kg^–1^.

##### On-Farm Survey

The exact locations of the on-farm demonstration strips as recorded by global positioning system (GPS) technology were lost during personnel changes. Hence, we describe in general terms their local landscapes and soil types (Prior, 1991). Most demonstration strips were in maize—soybean rotation fields in the Des Moines Lobe, Iowan Surface, and the Southern Iowa Drift Plain. These three landforms are characterized by Mollisols. A large majority of the soils within these landforms were formed under tallgrass prairie. While most surface soils in the Des Moines Lobe area were formed in glacial till, some soils of the Iowa Surface have overlying mantles of loess, and the Southern Iowa Drift Plain largely consists of loess surface soils over older glacial till deposits and are more eroded with deeper valleys than the other two landforms.

In 2011, six sites were also sampled in the Sand Hills region of north-central NE and south-central SD. Three were dryland, and three were irrigated due to low annual precipitation (508–570 mm yr^–1^). The six fields were located within Rock County NE, and Tripp County, SD. Soil orders in Rock County range from relatively young soil orders of Entisols and Inceptisols, to a few Mollisols (USDA Soil Conservation Service, 1985a). The Els-Valentine-Tryon soil association dominates the county. These are somewhat excessively to well-drained soils of sandy texture having low fertility and water-holding capacity. Tripp County, SD, has more diverse soils ranging in texture from fine sands to loams and clayey soils that are mostly of the Entisol and Mollisol soil orders (USDA Soil Conservation Service, 1979). The Millboro–Lakoma soil association is predominant, which has well-drained silty clays of moderate to low fertility.

### Field Designs and Management Practices

The Radcliffe field experiment in 2010 and 2011 and the Whitten field experiment in 2010 were each organized in randomized complete block designs. The plots were field-long treatment strips with maize rows at 76.2 cm spacing. Treatments in both fields compared different application timings of the previous formulation of the Yield Igniter^®^ humic product. This product was created through alkaline extraction of leonardite ore and contained about 30 g kg^–1^ of humic acid and 1.2 g kg^–1^ of fulvic acid (California Department of Food and Agriculture test). The rate of humic product application was 3.5 L product ha^–1^, following the recommendation of the manufacturer. The humic product was diluted with tap water to 94 L ha^–1^ and applied to the fields using standard agricultural sprayers, except for the in-furrow treatment at Conrad. In most cases, the nozzles were TeeJet XRC, and in some cases TeeJet drift guard (DG) nozzles were used, depending on the daily wind conditions, to maximize leaf interception and minimize wind drift. The pressure ranged from 207 to 310 kPa. At Radcliffe, the treatments compared a sole application at either preemergence, third leaf growth stage (V3), as defined by the leaf staging method that excludes the cotyledon leaf (Abendroth et al., 2011), or the sixth leaf stage (V6), compared to the unamended control. In the Whitten field, the treatments compared V3 and V6 applications against an unamended control. Both field experiments had four replications. In the 2011 Radcliffe field, one replicate was removed from the statistical analysis of the combine-measured grain yield because saturated soil conditions impaired the early season growth of maize in this replication. Each treatment strip contained 6 rows with 76.2-cm spacing in the Radcliffe field and 24 rows with 76.2-cm spacing in the 2010 Whitten field. Row length in both the fields was about 760 m. The 2011 Radcliffe plots were placed in the same locations as in 2010 by using the GPS and geographic information system technologies.

The Conrad field in 2013 contained two adjacent studies. Each was organized in a randomized split-plot design with four (north block) or five (south block) replicates. This design was intended to minimize data variability that could have arisen from soil drainage differences across this field. Main plot treatments in the north block compared three maize cultivars having relative maturity (RM) ratings (in days) of 100, 105, and 110, and subplots compared an unamended control to in-furrow application of the humic product with planting at the recommended rate. An adjacent south block had the same design except that the 105-day variety was omitted and the humic product was broadcast applied at the V5 growth stage. Row lengths in each Conrad block were about 62 m, and each plot had four maize rows of 76.2 cm spacing.

Thus, the timing of the humic product applications at the replicated field sites varied from in-furrow application with planting to V6. All other crop management practices across the entire fields were decided by the land managers, including cultivar, planting date, population density, fertilizer application rates, pest management, and harvesting practices. They followed management practices that are conventional for US maize production, and all fields received conventional tillage.

In each year of the on-farm survey, the Yield Igniter^®^ humic product was applied as demonstration strips in maize fields of collaborating farmers across much of central, southern, and northern Iowa, and also in 2011 at the six sites in SD and NE. The product was applied at post emergence through standard pesticide sprayers at early maize growth stages, not later than V6. The humic product was applied by the manufacturer in demonstration strips for all survey fields in 2009 and 2010 at their recommended rate of 3.5 L product ha^–1^, diluted with tap water to field-relevant volumes, while in 2011, some farmers performed the demonstration strips in their own fields. Following product application, the demonstration strips were not visited again and were left to farmer supervision until sampling time. While a few cooperating farmers participated in multiple years, their demonstration strips were not located on the same rows within those fields in all the years. Therefore, each paired comparison in each year represents a novel site location. Conventional crop management practices were followed and were selected by the managing farmer, including maize cultivar.

At crop physiological maturity in 2009, 2010, and 2011, about 30–35 production fields were hand-sampled for yield components across distinct regions in Iowa or adjacent states. All their data are presented here except for two fields in 2009, due to uncertain plot labels, and two fields in 2011, due to uncertain sample labels. In most cases, each field had only one demonstration strip. For the few fields where multiple demonstration strips were established, either one strip was randomly selected for sampling or all strips within each field were sampled and their means were calculated to represent that field.

### Plant and Soil Sampling

#### Maize Grain Yield Measurements by Combine and Weigh Wagon

For the central Iowa trials with replicated treatments strips, grain yield and moisture were recorded by mechanized combine. Yield monitor was used at the Whitten site for each field-long treatment strip, and we report the means of each treatment strip. At the Radcliffe and Conrad sites, weigh wagons were used to record grain mass and grain moisture (measured with a hand-held meter) along with yield monitor data that were hand-recorded for the field-long treatment strips. Weigh wagons were calibrated annually to the nearest 0.9 kg by their manufacturer, and then the weigh wagons were calibrated against the combine-yield monitors in each field prior to harvesting. Grain yield data from all sites were expressed as dry volume by adjusting them to the standard equivalent of 15.5% market moisture. For the on-farm survey, grain yield measurements by either combine-yield monitor or weigh wagon were not made available by any of the collaborating farmers. We chose not to confront their reluctance, as public and private sector advisors often discourage farmers from sharing their data.

#### Yield Components

For both years at the Radcliffe site and the on-farm surveys, plant samples were hand-harvested after maize kernels had achieved physiological maturity to determine yield components. They were collected in areas of uniform growth and similar soil type across all treatment strips and unamended controls for each field. Samples were collected from the Radcliffe field in the Nicollet soil for all treatment strips, and from the Whitten field in the Tama soil type.

Specifically, except the 2009 on-farm survey, a 1-m length section of one maize row was harvested by selecting an area of representative crop growth in each demonstration strip, then cutting seven evenly spaced healthy plants at ground level, and then separating the ears from the stover. This procedure was repeated nearby, within a limited number of maize rows outside the demonstration strip, to collect an unamended control sample while avoiding both edaphic differences and border effects. A more laborious method was used in the initial 2009 on-farm survey, by which a representative plant was sampled in each of eight consecutive rows at predetermined distances into each demonstration strip and, similarly, into the area of untreated plants immediately next to each demonstration strip. Soil samples were collected from the Radcliffe and Whitten fields in 2010 and from the on-farm survey in 2010 and 2011. Specifically, four soil cores were taken to the 15-cm depth with a 3.18-cm diameter probe in a row traversing the 1 m-hand-harvested section or (2009 survey only) in an untrafficked interrow at the final sampled plant, then composited within each treatment strip, and stored at 4^°^C until later analyses for nutrient contents and other soil properties.

All maize stover samples were oven-dried at 55^°^C in forced air dryer rooms, then immediately measured for oven-dry weights and mechanically shredded. Subsamples were taken from the shredded stover for later grinding through a Wiley mill (1 mm mesh screen) and then from a Cyclone mill (Udy Corporation, Fort Collins, CO) to a powder consistency. Maize ears were dried in 2009 in the same dryer rooms as were the stover, but in all subsequent years, they were placed in plastic mesh bags and hung for drying at ambient temperatures before being stored in airtight bins for subsequent measurements. Maize ear grains for the replicated field trials and the 2011 on-farm survey were later hand-shelled and passed through a mechanical seed counter for determining the 100-kernel weight. Total kernel weights of the hand samples were recorded, and kernel moisture was recorded by a moisture meter. Maize grain moisture content was also determined by a standard oven-drying method (ASAE, 1988). For all sites, the grain weight of each sample was then calculated and extrapolated to a hectare basis to present the grain yield as if each field were wholly homogenous. Given the soil type variability that can occur within field-long treatment strips, such extrapolations primarily express the yield response to the humic product only at the sampling site. Grain weights at all sites were expressed as dry volume by adjusting to the standard equivalent of 15.5% market moisture.

The lengths of air-dried cobs were measured for all hand-samples, and the cobs were then oven-dried for 3 days at 120^°^C and immediately measured for dry weight. The dried cob weights were then added to those of the 1-m stover samples to report total aboveground stover weight.

From the replicated trials at the Radcliffe and Whitten fields, and from the 2010 on-farm survey, subsamples of harvested grains were initially air-dried to no more than 100 g kg^–1^ moisture content and then stored in airtight plastic bags until later analysis for protein, oil, and starch contents using near-infrared spectroscopic procedures (Iowa Grain Quality Initiative, 2004).

Plant and soil samples were analyzed for predetermined sets of properties as offered by a commercial analytical laboratory. Total N analyses were performed on plant stover and grain through micro-Kjeldahl digestion and colorimetric determination of the extracted N content. Plant stover and grain analyses for all other nutrients (P, K, Mg, Ca, S, Zn, Mn, Cu, Fe, and B) were performed using wet digestion in nitric acid with 30% hydrogen peroxide and determination by inductively coupled plasma-mass spectrometry. Plant Na and Al were also measured, but their results are not reported due to their erratic, and at times absent, concentrations and relatively low precision of analysis.

Methods for measuring soil extractable nutrients, pH, buffer pH, organic matter, and cation exchange capacity followed the Recommended Chemical Soil Test Procedures for the North Central Region, Publication No. 221 Revised (Denning et al., 1998). Soil pH was determined in a 1:1 (w:v) slurry in water, and buffer pH by the Sikora Buffer method. Soil organic matter content was determined through loss on ignition. Available soil P was determined colorimetrically from a Bray 1 extraction (Bray and Kurtz, 1945). Available soil cations were extracted with 1 M ammonium acetate and analyzed by inductively coupled plasma-mass spectrometry.

In the Radcliffe and Whitten field trials, all maize leaves were destructively measured for leaf area measurement on selected plants in areas of uniform growth. Triplicate sets of three plants were marked at the V5 or V6 crop stage for three in-field samplings. The first leaf area measurement was at the V5 or the V6 growth stage. At the same time, flagging tape was used to mark the internode between the V6 and V7 leaves of the other two plant sets. One of these sets was later used for the second measurement of the leaf area at the V11 or V12 growth stage. Flagging tape was also used then to mark the internode between the V11 and V12 leaves of the final plant set for the third leaf area measurement soon after full tassel (transition from vegetative to reproductive growth stage). For each leaf, its length and maximum width were measured to calculate leaf area by the method developed by Montgomery (1911) using the following equation:


(1) Length⁢(cm)×Maximum⁢Leaf⁢Width⁢(cm)×0.75=LeafArea(cm)2

Total plant leaf area was the sum of the areas from all the leaves of each plot. The first two leaves of each plant were often lost already at the first leaf area measurement, due to senescence or physical damage.

### Statistical Analyses

All experimental data from the central Iowa trials with replicated treatment strips were analyzed by ANOVA *via* the Proc Mixed procedure of SAS Version 9.2 software (SAS Institute, 2010) with randomized complete block or split-plot design programs to examine main plot treatment, split-plot treatment, and interaction effects. Paired *t*-tests were conducted by the least significant difference method. At the Conrad site, the cultivar—humic product interaction terms for both the blocks were insignificant (*P* > 0.10) and are not shown (Table 2).

**TABLE 2:** Maize grain yield measured by combine for replicated field trials at Radcliffe, Whitten, and Conrad with the application of the humic product at preemergence, third leaf stage (V3), fifth leaf stage (V5), or the sixth leaf stage (V6).

2010 Radcliffe			
**Humic treatment**	**Maize grain yield (Mg ha^–1^)**	**Probability of statistical significance**	

		Humic main plot	0.010
Control	13.20		
Pre-emergence	13.49	Paired *t*-test vs. Control	0.012
V3	13.54	Paired *t*-test vs. Control	0.002
V6	13.58	Paired *t*-test vs. Control	0.005

**2011 Radcliffe**			

		Humic main plot	0.125
Control	12.54		
Pre-emergence	12.89	Paired *t*-test vs. Control	0.033
V3	12.76	Paired *t*-test vs. Control	0.126
V6	12.83	Paired *t*-test vs. Control	0.066

**2010 Whitten**			

		Humic main plot	0.283
Control	13.90		
V3	14.05	Paired *t*-test vs. Control	0.227
V6	14.09	Paired *t*-test vs. Control	0.152

**2013 Conrad North block** ^a^			

		Varietal main plot	0.038
100 RM^b^	9.95		
105 RM	11.17	Paired *t*-test vs. 100 RM	0.017
110 RM	10.93	Paired *t*-test vs. 100 RM	0.043
Control	10.48		
Humic at Planting	10.90	Paired *t*-test vs. Control	0.064

**2013 Conrad South Block** ^a^			

		Varietal main plot	0.037
100 RM	10.39		
110 RM	11.13	Paired *t*-test vs. 100 RM	0.037
Control	10.65		
Humic at V5	10.88	Paired *t*-test vs. Control	0.212

*^a^Cultivar—humic product interaction terms for both blocks were insignificant (P > 0.10) and are not shown.*

*^b^Relative maturity rating (estimated in day units).*

For the on-farm survey, we used SAS Version 9.2 (SAS Institute, 2010) to perform ANOVA for evaluating humic product application as the independent variable and the difference between humic-treated and control plant samples at each site to calculate each of the dependent variables: maize yield components and nutrient concentrations in grain, stover, and soil. Each site was treated as a single replication. The site factor was treated as a random effect in a two-factor ANOVA comparing the control and humic treatment group (Factor 1) and three specific years of 2009–2011 (Factor 2), these two factors being treated as fixed effects, and then examining the interaction between the group and the year.

Field crop responses to humic products can in cases be modest, but they can also change gradationally with local environmental conditions (Olk et al., 2021). For example, Olk et al. (2021) found that maize growth responses to a humic product were frequently weakly positive across three soil types in four growing seasons, but they were much more likely to reach statistical significance (*P* < 0.10) in droughty conditions. Adhering to a preselected level of significance is somewhat a subjective decision, and useful information can be lost regarding the patterns of gradational responses. Therefore, we report individual levels of significance (P) for key plant growth parameters to depict gradational responses more accurately. At the same time, we summarize large datasets of plant and soil parameters having secondary value by setting a benchmark level of significance at *P* = 0.10.

## Results

### Replicated Field Trials in Central Iowa

#### Mechanized Grain Yield

In the 2010 Radcliffe field, all three timings of product application provided for grain yields (measured by weigh wagon) that were 0.29 to 0.38 Mg ha^–1^ (2 to 3%) greater than the grain yield of the unamended control (Table 2). The main plot treatment was highly significant (*P* = 0.0095). When comparing each treatment with the control by paired *t*-tests, all differences ranged from significant (*P* < 0.05) to highly significant (*P* < 0.01). In 2011, the three treatments similarly provided yield increases of 0.22–0.35 Mg ha^–1^ (2–3%). With only three field replications in 2011, the main plot treatment approached benchmark significance (*P* = 0.125). Paired *t*-tests for individual treatments found levels of significance varying from 0.033 to 0.126.

In the 2010 Whitten field, the two application timings increased the grain yield (measured by combine yield monitor) by 0.15 and 0.19 Mg ha^–1^ (1%) more than the control in this traditionally high-yielding field (Table 2). The main plot treatment was insignificant (*P* = 0.283). Paired *t*-tests were insignificant (*P* = 0.227 and 0.152).

In the 2013 Conrad field northern block, maize grain yield was significantly greater (*P* = 0.038) for the 105-RM and 110-RM varieties than for the 100-RM variety (Table 2). At the subplot level, humic product application increased the grain yield across all three maize varieties by 0.42 Mg ha^–1^ (4%), which was significant at *P* = 0.064. Paired *t*-tests found significant (*P* < 0.05) differences among varieties when comparing the 100-RM variety against each of the longer-duration varieties. In the Conrad field southern block, the 110-RM maize variety again had significantly (*P* = 0.037) greater grain yield than did the 100-RM variety (Table 2). Humic product application again provided for a numeric increase in the grain yield above the unamended control across both varieties, but only by an insignificant amount of 0.23 Mg ha^–1^ (2%, *P* = 0.212) (Table 2). Summarizing the replicated field trials, combine-measured grain yield increased numerically with humic product application in all five site—years, and its magnitude was generally larger in those site—years where the control had relatively lower grain yields. Thus, the yield response was larger in the lower-yielding Radcliffe and Conrad North fields but was of the smallest magnitude in the high-yielding Whitten field. These variable responses, in turn, affected the degree of statistical significance of the yield response for each site—year. In all cases, they were modest proportional increases.

#### Yield Components at the Radcliffe Field

In 2010, grain weights of the hand-collected samples, as extrapolated to a hectare basis, increased numerically with humic product application by 0.36 to 1.18 Mg ha^–1^ (2–7%), and the increases were largest with the earlier application (Table 3). But the main plot treatment was insignificant (*P* = 0.70), and paired *t*-tests between the control and each application time also showed no significant differences (*P* > 0.10). In 2011, for the same field, grain weights again increased numerically with humic product application, by 0.44 to 1.31 Mg ha^–1^ (3–8%), and the increases were, again, largest with the earlier application. In this year, the main plot treatment approached benchmark significance (*P* = 0.156), and paired *t*-tests showed a significant difference (*P* = 0.04) between the V3 application and the control.

**TABLE 3:** Maize yield components at the Radcliffe site in 2010 and 2011.

Humic treatment		Probability (P) of statistical significance	
**2010 Grain weight (Mg ha**^–^**^1^)**			

		Humic Main plot	0.697
Control	16.50		
Pre-emergence	17.68	Paired *t*-test vs. Control	0.286
V3	17.31	Paired *t*-test vs. Control	0.460
V6	16.86	Paired *t*-test vs. Control	0.740

**2011 Grain weight (Mg ha**^–^**^1^)**			

		Humic Main plot	0.156
Control	16.25		
Pre-emergence	16.70	Paired *t*-test vs. Control	0.403
V3	17.57	Paired *t*-test vs. Control	0.037
V6	17.00	Paired *t*-test vs. Control	0.183

**2010 Cob length (mm)**			

		Humic Main plot	0.969
Control	158.6		
Pre-emergence	159.9	Paired *t*-test vs. Control	0.797
V3	157.6	Paired *t*-test vs. Control	0.833
V6	159.2	Paired *t*-test vs. Control	0.945

**2011 Cob length (mm)**			

		Humic Main plot	0.110
Control	160.1		
Pre-emergence	164.4	Paired *t*-test vs. Control	0.128
V3	165.3	Paired *t*-test vs. Control	0.074
V6	167.2	Paired *t*-test vs. Control	0.026

**2010 One hundred-kernel weight (g 100 kernel**^–^**^1^)**			

		Humic Main plot	0.108
Control	25.13		
Pre-emergence	27.15	Paired *t*-test vs. Control	0.022
V3	25.70	Paired *t*-test vs. Control	0.460
V6	25.90	Paired *t*-test vs. Control	0.323

**2011 One hundred-kernel weight (g 100 kernel**^–^**^1^)**			

		Humic Main plot	0.722
Control	31.66		
Pre-emergence	31.34	Paired *t*-test vs. Control	0.495
V3	31.77	Paired *t*-test vs. Control	0.804
V6	31.39	Paired *t*-test vs. Control	0.556

**2010 Stover weight (Mg ha^–1^)**			

		Humic Main plot	0.650
Control	11.80		
Pre-emergence	12.24	Paired *t*-test vs. Control	0.519
V3	11.49	Paired *t*-test vs. Control	0.651
V6	11.50	Paired *t*-test vs. Control	0.669

**2011 Stover weight (Mg ha^–1^)**			

		Humic Main plot	0.322
Control	12.76		
Pre-emergence	13.66	Paired *t*-test vs. Control	0.295
V3	14.28	Paired *t*-test vs. Control	0.099
V6	13.13	Paired *t*-test vs. Control	0.653

In 2010, all application treatments had non-significant effects (*P* > 0.10) on cob length, as determined by paired *t*-tests with the control. In 2011, however, both the V3 and V6 applications of the humic product caused significant (*P* = 0.074 and *P* = 0.026, respectively) increases in the cob length. The preemergence application caused a slightly weaker yet still positive response that approached benchmark significance (*P* = 0.13), and the overall main plot treatment similarly approached benchmark significance (*P* = 0.110).

For 100-kernel weight in 2010, the main plot treatment was significant at *P* = 0.108, and preemergence application in 2010 caused a significant positive (*P* = 0.022) response, as determined by a paired *t*-test with the control. For the other treatments in 2010 and all treatments in 2011, the paired *t*-tests showed non-significant (*P* > 0.10) differences from the control. All differences from the control in 2011 were 1% or less.

In 2010, all application treatments had non-significant effects (*P* > 0.10) on stover weight. In 2011, the V3 application increased stover weight by 12% (*P* = 0.099). The preemergence treatment increased stover weight by 7% but was not significant (*P* = 0.30). Across both the years, the only cases of humic product application that significantly (*P* < 0.10) affected the grain content of protein, oil, or starch were found in 2010 (data not shown). Specifically, V3 application increased (*P* = 0.085) the protein content, and V6 application increased (*P* = 0.099) the starch content. No numeric trends were apparent in the remaining results.

For grain or stover concentrations of N, P, K, Mg, Ca, S, Zn, Mn, Cu, Fe, and B in either year, the only nutrients that significantly responded (*P* < 0.10) to any humic product treatment were decreases in stover Mg (*P* < 0.050) with V3 application and stover Zn (*P* < 0.097) with V6 application in 2010, and an increase in grain Mg (*P* = 0.041) with V3 application (data not shown) in 2011. No numeric trends were apparent in the remaining results (data not shown).

Of the soil properties measured in the 2010 Radcliffe and Whitten fields (soil organic matter content, pH, buffer pH, cation exchange capacity, total N, extractable P, K, Mg, Ca, S, Fe, Zn, Mn, Cu, and B), the humic product effects were significant (*P* < 0.10) at Radcliffe for only increased extractable Cu (*P* = 0.065) and at Whitten for only increased extractable Mn (0.088) (data not shown). Paired *t*-tests for individual treatments found significant (*P* < 0.10) increases at the Radcliffe field for only Cu with preemergence (*P* = 0.018) and V3 applications (*P* = 0.027) and at the Whitten field for only K (*P* = 0.083), Mn (*P* = 0.016), and Cu (*P* = 0.049) with the V3 application. Only occasional numeric trends were apparent in the remaining results, in no meaningful pattern (data not shown).

#### Leaf Area

At the Radcliffe site in both 2010 and 2011 and at the 2010 Whitten field, all humic product treatments provided significantly (*P* < 0.10) greater total leaf area than did the unamended control (Table 4). The increases reached as high as 12% for the preemergence application at the 2011 Radcliffe site. Main plot humic treatment effects were also significant for the 2010 Radcliffe (*P* = 0.0138), 2011 Radcliffe (*P* = 0.0701), and 2010 Whitten (*P* = 0.0103) sites.

**TABLE 4:** Total leaf area and level of statistical significance (P)^#^ by individual leaf areas for three replicated field trials.

	2010 Radcliffe				2011 Radcliffe				2010 Whitten		
Treatment	Control	Pre-emerge	V3	V6	Control	Pre-emerge	V3	V6	Control	V3	V6
Total area (cm^2^)	7040	7516	7341	7282	6625	7401	7271	7389	6694	7427	7362
*P*		*0.002*	*0.024*	*0.057*		*0.025*	*0.048*	*0.026*		*0.010*	*0.009*

Leaf	Level of statistical significance (P) by individual leaf										

V1	–	–	–	–	–	–	–	–	–	–	–
V2	–	–	–	–	–	0.301	**0.018**	0.517	–	–	–
V3	–	0.466	0.568	0.923	–	0.804	0.575	0.673	–	0.446	0.929
V4	–	0.412	**0.039**	**0.055**	–	0.828	0.604	0.629	–	0.736	0.192
V5	–	0.786	0.152	0.258	–	0.501	0.278	0.418	–	0.871	0.460
V6	–	0.758	**0.096**	**0.037**	–	0.850	0.655	0.700	–	0.882	0.304
V7	–	0.906	0.681	0.214	–	**0.004**	**0.001**	**0.004**	–	0.984	0.491
V8	–	0.345	0.373	0.970	–	**0.004**	**0.008**	0.190	–	0.540	0.612
V9	–	**0.072**	**0.084**	0.661	–	0.192	0.837	0.458	–	0.939	0.930
V10	–	**0.006**	**0.034**	0.367	–	0.228	0.479	0.728	–	0.947	0.715
V11	–	**0.005**	**0.019**	0.119	–	**0.033**	0.760	0.325	–	0.415	0.272
V12	–	**0.002**	**0.021**	**0.049**	–	**0.014**	0.228	**0.006**	–	0.406	**0.063**
V13	–	**0.004**	**0.055**	**0.025**	–	**0.044**	0.119	**0.012**	–	**0.012**	**0.007**
V14	–	**0.010**	0.165	**0.092**	–	0.139	0.144	**0.059**	–	**0.027**	0.174
V15	–	**0.034**	0.368	**0.006**	–	**0.054**	**0.029**	**0.017**	–	**0.014**	**0.039**
V16	–	0.119	0.372	**0.095**	–	**0.042**	0.111	**0.033**	–	0.260	0.458
V17	–	0.408	0.538	0.323	–	**0.057**	**0.091**	**0.036**	–	0.167	0.286
V18	–	**0.037**	0.295	**0.023**	–	0.136	0.125	**0.047**	–	0.102	**0.058**
V19	–	0.675	0.696	0.640	–	–	–	–	–	0.152	0.164
V20	–	0.828	0.656	0.427	–	–	–	–	–	–	–

*Significant values (P < 0.100) for individual leaves are shown in bold font.*

*^#^Statistical significance for total leaf areas and individual leaves determined by paired t-tests against the control.*

**FIGURE 1:**
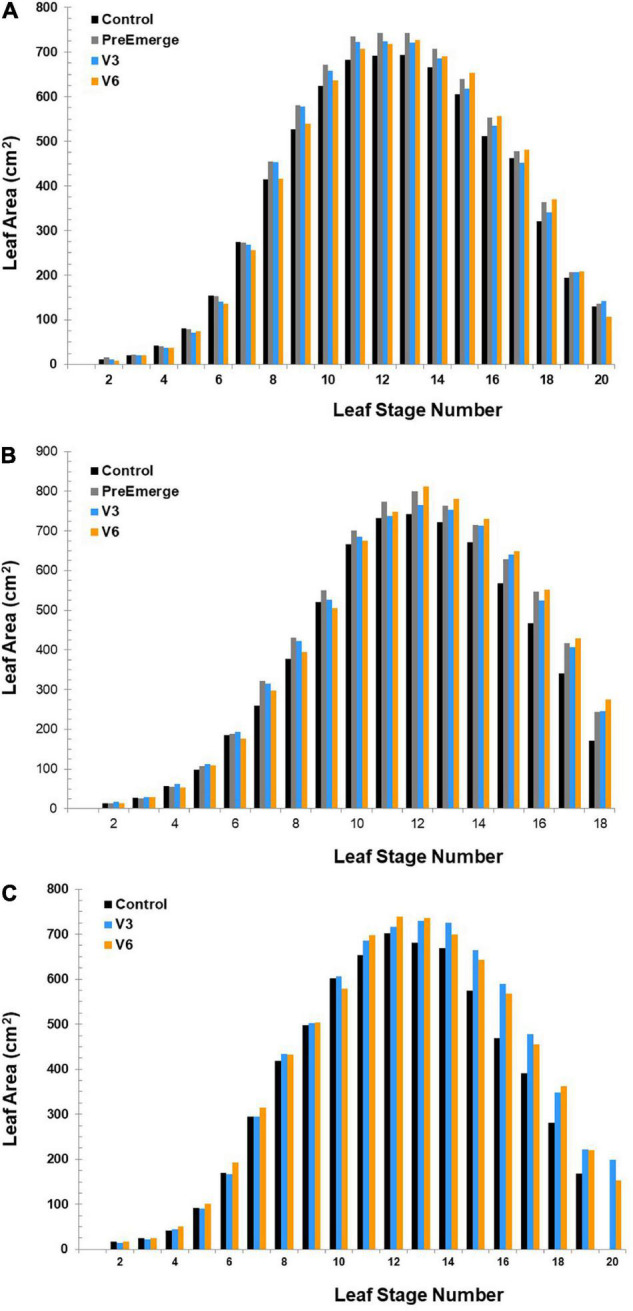
Leaf area for the **(A)** 2010 Radcliffe field, **(B)** 2011 Radcliffe field, and **(C)** 2010 Whitten field by individual stage and time of humic product application. The V3 time of humic application is the third leaf vegetative growth stage, and V6 is the sixth leaf vegetative growth stage.

Leaf area by individual leaves showed infrequent positive responses to the humic product by the earliest leaves; we attribute them to random variation among plots when selecting healthy plants at an early growth stage. Positive responses to humic product application became consistent no earlier than the 7th leaf for the preemergence and V3 applications and the 10th or 11th leaf for the V6 applications (Figures 1A–C). The increases became consistently significant (*P* < 0.10) for the Radcliffe preemergence application at about the 7th leaf (2011) or 10th leaf (2010) and remained significant for most leaves through the 17th or the 18th leaf (Table 4). The V3 application showed a weaker response but of comparable timing. Significant increases for the V6 application became consistent at all sites starting at about the 12th leaf and remained significant for most leaves until the 15th (Whitten) or 18th leaf (Radcliffe). Thus, the benefit to leaf area of the V6 application was somewhat delayed compared to those of earlier applications. Numeric trends suggested that leaf area growth might have been depressed briefly after the foliar applications compared to the control, specifically for both the V3 and V6 applications at the 2010 Radcliffe site, V6 application at the 2011 Radcliffe site, and V3 application at the 2010 Whitten site (Figures 1A–C). This decrease reached statistical significance (*P* = 0.037 and *P* = 0.004, respectively) for V6 applications in both years at Radcliffe.

### On-Farm Survey

The vast majority of the sites were located on the Des Moines Lobe, with smaller numbers of farms on the Iowa Surface and very few on the Southern Iowa Drift Plain. Maize responses did not clearly differ among these three geomorphic surfaces; therefore, all Iowan sites are presented as one set. The 2011 NE and SD sites did differ clearly from the Iowa sites, so we present the 2011 results both as one complete set and also with the NE and SD sites separated from the Iowa sites.

In multi-year combined statistical analyses for the on-farm survey data, agronomic yield components showed a statistical significance. However, the year factor was significant for all measures. This was not surprising, given that weather patterns substantially affect crop growth and soil nutrient availability. In addition, for each year, many sample sites were not in the same fields as in the previous years. Therefore, we initially present these on-farm survey measures by individual year. Humic product—year interactions were insignificant (*P* > 0.10) for all plant and soil measurements, which are not shown.

**FIGURE 2:**
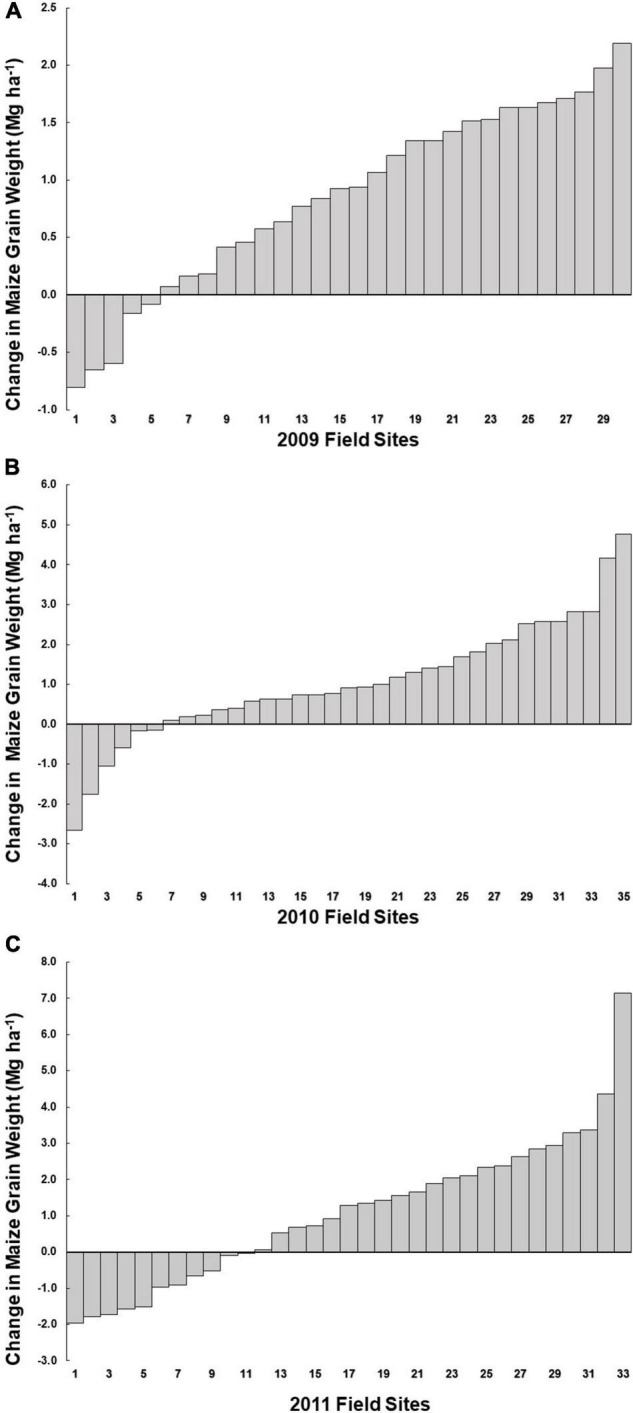
Maize grain weight response to humic product application compared to an adjacent unamended control at on-farm survey sites in **(A)** 2009, **(B)** 2010, and **(C)** 2011. In the 2011 survey, Nebraska (NE) sites are numbered 10, 25, and 33, and South Dakota (SD) sites are numbered 14, 29, and 32.

In each year, grain weight per hectare, as extrapolated from the yield component samples, increased numerically with humic product application for the vast majority of farms. In 2009, the grain weight increase occurred at 25 of 30 farms, or 83% (Figure 2A). Mean grain weight across all 30 farms increased with the product application by 5.7%, or 0.98 Mg ha^–1^ (*P* < 0.0001) (Table 5). In 2010, grain weight was numerically greater for 29 of 35 farms, or 83% (Figure 2B), and grain weight increased across the 35 farms with product application by 6.7%, or 1.05 Mg ha^–1^ (*P* = 0.0002). In 2011, 22 of 33 farms (67%) had numerically greater grain weight with humic product application (Figure 2C). The coarser textured, dryland production and irrigated sites in SD and NE were among the more responsive sites to product application in 2011, averaging 22% increase, or 2.9 Mg ha^–1^ (*P* = 0.041), while for the Iowa 2011 sites, the mean increase was 4.2%, or 0.68 Mg ha^–1^ (*P* = 0.043) (Table 5). Mean grain weight in the unamended controls of the SD and NE sites was only 81% of the mean for the Iowa controls. Across all the 3 years, grain weight increased with humic product application in 76 of 98 cases (78%). We do not propose a single explanation for the negligible or negative responses for 22 of the 98 cases, other than the observation that a few sites were excessively wet, and limited evidence suggests humic product efficacy is sharply impaired in excessively wet soils (Olk et al., 2021).

**TABLE 5:** Maize yield components for individual years of the on-farm survey.

Humic treatment	Grain weight (Mg ha^–1^)	Cob length (cm)	Stover weight (Mg ha^–1^)	100-kernel weight (g 100 kernel^–1^)
	2009			
Control	17.15	17.23	12.82	–
Humic treated	18.13	17.62	13.56	–
*F*-test *P*-value	*<0.001*	*0.001*	*0.003*	–
	2010			
Control	15.77	16.06	11.20	—
Humic treated	16.82	16.62	12.02	—
*F*-test *P*-value	*<0.001*	*0.003*	*0.001*	–
	2011—All sites			
Control	15.52	16.30	11.27	29.94
Humic treated	16.61	16.75	11.90	30.60
*F*-test *P*-value	*0.004*	*0.003*	*0.016*	*0.170*
	2011—Nebraska and South Dakota sites only			
Control	12.95	16.79	9.08	24.52
Humic treated	15.85	17.65	10.87	27.20
*F*-test *P*-value	*0.041*	*0.031*	*0.053*	*0.118*
	2011—Iowa sites only			
Control	16.09	16.19	11.76	31.15
Humic treated	16.77	16.55	12.13	31.36
*F*-test *P*-value	*0.043*	*0.032*	*0.129*	*0.639*

*Number of observations was 98 for grain weight, 95 for cob length and stover weight, and 33 for 100-kernel weight (2011 only).*

**FIGURE 3:**
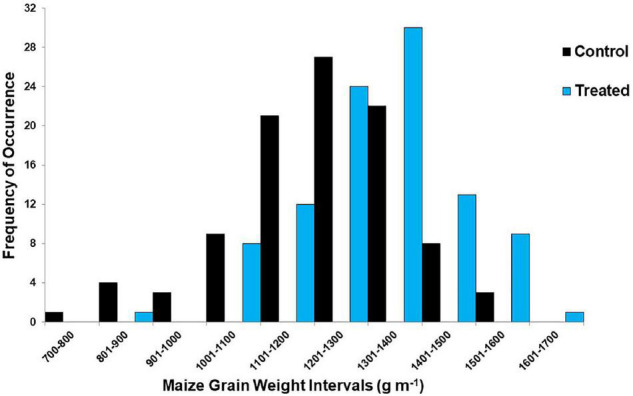
Frequency of occurrence for 10 intervals of maize grain weight for the humic product vs. unamended control treatments at 98 locations in the on-farm survey, 2009–2011.

To combine the data across all the 3 years, grain weights from the 8 plants collected from the 2009 plots were adjusted to the 7-plant basis of the 2010 and 2011 seasons. The adjusted data from 2009 to 2011 were analyzed collectively for their distribution across 10 intervals of grain weights for the humic product-treated samplings and separately for the controls (Figure 3). The grain weights from both the unamended plots and also the humic-treated plots occurred mostly in the same ranges of grain weights; the humic product scarcely increased the grain weight beyond the maximum values achieved in the control plots. Instead, product application led to greater proportions of the medium- and high-grain weights and lesser proportions of the lower-grain weights. Mean mass across all 98 paired comparisons was 1.23 kg m^–1^ for the control and 1.31 kg m^–1^ for the treated plots, a highly significant (*P* < 0.001) increase of 0.08 kg m^–1^ (6.5%), or 1.05 Mg ha^–1^. In short, humic product application significantly increased the grain weight, mostly by increasing what would have been lesser grain weights to more moderate weights.

**FIGURE 4:**
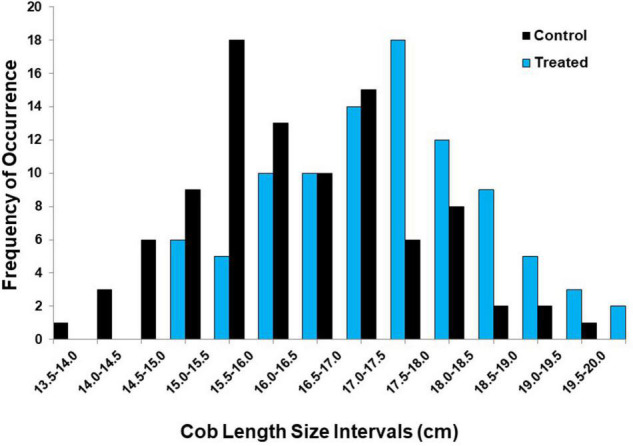
Frequency of occurrence for 13 cob length intervals for the humic product vs. unamended control treatments at 98 locations in the on-farm survey, 2009–2011.

Similar to grain weights, humic product application did not alter the range of cob lengths compared to that of the control for 95 of the same 98 farms across all the 3 years (Figure 4). Instead, humic product application again caused greater proportions of the medium-length and long cobs, with smaller proportions of the shorter cobs, compared to the control plots. With humic product application, cob length for all 95 farms increased by 3% from 16.5 to 17.0 cm, which was highly significant (*P* = 0.0053). Cob length also increased significantly for each of the 3 years from 2009 to 2011 (*P* = 0.0005, 0.0026, and 0.0033, respectively, Table 5). Like grain weight, cob length at the irrigated sites in NE and SD in 2011 responded especially well, with a 5% increase (*P* = 0.031). Using calculations presented by Nielsen (2018), our observed increase in the cob length of 0.5 cm across all 95 farms translates into an increase in the grain weight of about 0.5 Mg ha^–1^, presuming a grain diameter of 0.4 cm, 14 rows of grain per cob, and complete kernel filling. Thus, the increased cob length with humic product application accounted for about half of the measured increase in the grain weight. The remaining yield increase might be partially attributable to a more complete grain filling of the cob, which we observed routinely.

Of the other yield components, the stover mass responded similarly as did the grain weight. Across all the 3 years (*n* = 98), it increased significantly (*P* = 0.002) with humic product application by 6.2% (data not shown). For each of the 3 years, its increases were in the sequence of 5.8% (*P* = 0.002), 7.3% (*P* = 0.0009), and 5.6% (*P* = 0.016) (Table 5). For the 2011 dryland and irrigated sites in NE and SD, the increase in stover mass was a vigorous 20% (*P* = 0.053). The 100-kernel weight was recorded only in 2011. Humic product application caused a 2% increase in the 100-kernel weight across all sites, which approached benchmark significance (*P* = 0.17). The NE and SD sites showed a numerically more vigorous response that more closely approached benchmark significance (*P* = 0.12). Three parameters of grain quality were measured only in 2010. Their responses to humic product application were generally insignificant for oil content (*P* = 0.162), starch content (*P* = 0.54), and protein content (*P* = 0.90) (data not shown). Field observations found that the number of developed ears never changed with the humic product application. Frequent checks in 2009 found no effect of the humic product on the number of kernel rows on each ear (data not shown).

Humic product application did not significantly (*P* > 0.10) affect the total concentrations of N, P, K, Mg, Ca, or Fe in either the grain or the stover (data not shown). Neither did it statistically affect the amounts of any of these same nutrients extracted from soil either in 2009 or 2010 or across both the years, nor soil organic matter content, pH, buffer pH, or cation exchange capacity (data not shown). Similarly, concentrations of S, Zn, Mn, Cu, and B as total plant nutrients or extractable soil nutrients showed no numeric trends with humic product application (data not shown).

## Discussion

A major knowledge gap constraining the widespread use of humic products concerns their reliability over time and space in benefiting crop growth. Humic products do not appear to promote crop growth in all situations, given the variable results reported to date (Olk et al., 2021). Thus, the need arises to determine whether there is a predictable pattern in when and where the humic products improve crop growth and provide economically viable returns. As the first step, this study provided a wider scope of field settings for measuring agronomic benefits to maize production in the US Midwest than has been presented previously.

First, our results show that the recommended rates of humic product application have the capacity to boost maize growth in field conditions, even in a high-yielding region like central Iowa. In all eight treatment—year combinations of the replicated field trials, where leaf area was measured, total leaf area increased significantly (P < 0.10) with humic product application (Table 4), indicating that the humic product created improved growing conditions for the crop (Eik and Hanway, 1965). Statistically significant (*P* < 0.10) responses by individual leaves occurred mostly in the second half of vegetative growth, indicating that these growth stages might occur at the time of maximum product effect on plant processes, at least for the application times used in this study.

The enhancement of crop growth leading to increased grain yield may well depend on multiple factors, especially on the severity of other yield constraints. Among the replicated field trials, combine-measured grain yield responded most to product application at the slightly lower yield levels of the Radcliffe and North Conrad fields. The most productive field, Whitten, in the favorable 2010 growing season, showed only a slight numeric yield response to the humic product. The South Conrad field also showed only a slight yield response. This field tended to be seasonally wet, and abnormally high precipitation amounts fell in the 2013 early season. Olk et al. (2021) postulated that humic product benefits to upland crop growth are diminished in seasonally wet soils. Overall, all sites gave numerically positive yield increases.

Among the results from the on-farm survey of hand-harvested plant samples, especially notable is the frequency of grain weight increases in all 3 years of the on-farm survey, reaching 78% of all cases. The frequency of numeric increases was high in each year, varying only between 67 and 83% of all cases in each of the 3 years. Thus, although the three growing seasons varied somewhat in their precipitation patterns and perceived drought stress, no consistent effect of the weather variability was observed. A wide range of responses across the farms was recorded within each year, but like the replicated field trials, the responses were numerically mostly positive. Grain weight responses to the humic product were not clearly different among the three Iowa landforms—Des Moines Lobe, Iowan Surface, and the Southern Iowa Drift Plain. Among the most responsive sites were the dryland and irrigated sites in the sandier, less fertile soils of NE and SD of 2011, where maize growth in the controls was clearly less than in the controls in the more fertile Iowan soils. Many of the individual grain weight responses in the on-farm survey would not be statistically significant in a study having limited replication. Similarly, the maize growth responses in the replicated field trials were often weak statistically. Yet with the high number of field replicates in this on-farm survey, these differences became highly significant (*P* < 0.001). Hence, inconsistent field evaluations of humic products might in cases be due to an inadequate number of field replications to discern a potentially modest benefit. The number of recommended replicates may well vary by study, depending on the crop type, soil type(s), and general yield level in the local region.

This study presents both the replicated field trials and the on-farm survey to highlight their common findings. An extensive on-farm survey carries inherent research limitations and is presented here as supplementary to the replicated field trials. Researchers did not perform or supervise the application of the humic product at the survey sites, although we collected all plant and soil samples. Mechanized grain yield estimates were not made available by the farmers; hence, the sampled area was much smaller than a field-long strip. Location of the yield component sampling within each field involved some judgment, and as previously noted, the obtained grain weights represent maize response only from the sampled area, and not from the entire field.

Yet the consistency in results gained from both the replicated field trials and the on-farm survey merit noting. The replicated field trials and the on-farm survey shared the findings of generally positive grain responses to the humic product. Similarly, Olk et al. (2021) reported mostly positive responses of maize-combine grain yields, yield components, and total leaf area to a different humic product across 4 years and two landscape positions within two central Iowan fields. Annual precipitation patterns varied more in that study than the present one, ranging from severe drought to nearly ideal conditions. Olk et al. (2021) reported grain yield responses that were statistically significant only in droughty conditions. Both the studies suggest that humic products can promote crop growth in field conditions, as represented by the leaf area data found here, but whether that promotion leads to significantly greater economic yield depends on additional localized factors.

Combine-measured grain yields in the replicated field trials averaged an increase of about 0.3 Mg ha^–1^ with humic product application, while hand-sampled yield components from both years at the Radcliffe site increased by about 0.8 Mg ha^–1^, and the mean yield increase for hand-sampled plants of the on-farm survey reached 1.0 Mg ha^–1^. Three apparent explanations for this discrepancy between combine and hand-sampling are that, first, the hand-sampling avoided areas within a field where maize growth was visibly stunted by local environmental conditions, including potholes and eroded soils. Second, hand-sampling targeted plants of healthy growth, thus avoiding plants whose growth was limited by disease, insects, wind damage, or irregular plant spacing. Third, maize grain loss with hand-sampling was essentially non-existent, while with mechanized harvesting, ears can be dropped. For all these potential explanations, hand-sampling served to avoid conditions that might diminish the observed plant capacity to respond to the humic product. In contrast, mechanized combining would have harvested such growth-limited plants, possibly lowering the overall crop responsiveness to the humic product. It is informative to present both types of grain yield responses, as they show the potential and also the actual crop responses to humic product application in field conditions.

Cob length was a responsive yield component to humic product application in the on-farm survey, and it was also responsive in one of the 2 years at Radcliffe. In the maize field study by Olk et al. (2021) discussed previously, cob length was the yield component that was mostly responsible for increases in grain yield with humic product application. Potential ear length is determined by at least V15 and maybe as early as V12 (Strachan, 2004), and it is strongly affected by environmental stresses. Favorable leaf area responses to the humic product in these growth stages (Figures 1A–C) indicate that with humic product application the plant perceived better growing conditions involving less stress than in the control, thus possibly promoting the development of longer ears.

In both the studies, humic product application led to greater responsiveness of the smaller cobs in cob length and grain weight than that of the larger cobs, creating more homogenous ear sizes. This trend is seen in the shift of those measures in class size distributions (Figures 3, 4). As discussed by Olk et al. (2021), greater responsiveness of the smaller ears indicates that the primary benefit of the humic product may be to help smaller plants better compete with their larger neighbors for growth requirements. This hypothesis can be phrased as an example of stress alleviation, which would match the statements made by Calvo et al. (2014) that humic product benefits to plant growth often consist of alleviating environmental stresses. Also, the consistency of these findings across both maize studies, despite the use of different humic products, provides some evidence to the fact that the hand-sampling in this on-farm survey provided plausible results.

We presented limited data on the response of 100-kernel weight to the humic product. Its response was positive in the 2010 Radcliffe treatments and in the sole year when it was measured for the on-farm survey. These responses were mostly weak statistically. Such results are consistent with the results of Olk et al. (2021), who also found that the 100-kernel weight responses to humic product application were frequently positive but statistically often insignificant. Hence 100-kernel weight does not appear to be the primary driver for grain yield increase with humic product application in central Iowa, which has fertile soils and generally favorable climate for crop production. But it may well provide a secondary contribution. For regions where soils are less fertile and water deficits are more common, yield component responses to a humic product application could vary from those of this study, or they could be generally more pronounced, as we observed for the six NE and SD sites.

Further evidence that humic product use affected basic processes of plant growth was suggested by the beginning and end dates of the grain-filling period; that is, ear pollination and physiological maturity as represented by necrosis of kernel tips (“black layer”), respectively. In four 2009 maize production fields, pollination dates were scored visually as complete darkening of the ear silks through necrosis for humic product treated vs. control plots. In all fields, silk darkening (and hence pollination) occurred on average 3 days earlier for the treated plots than for the control. Yet in the three fields that were monitored at the end of the season, physiological maturity with product application was delayed by about 6 days. Thus, the grain-filling period was extended by about 10% (Abendroth et al., 2011) through both an earlier start and delayed finish. We speculate that extended grain-filling time was prompted by the previous development of larger ears, which would require more time for optimal grain-filling.

Our nutrient uptake data showed no consistent responses to humic product application for any nutrient concentration in either the grain or stover. Olk et al. (2021) also reported a similar lack of consistent nutrient response for maize growth in Central Iowa. Soil properties showed no consistent effect of the humic product on soil nutrient availability, although most plots received only 1 year of humic product use. Olk et al. (2021) reported similar results. These findings speak against a common industry belief that humic products enhance soil nutrient availability and instead point toward a plant-based mechanism for improved crop growth.

In summary, this study reported numerically positive responses of maize growth and grain yield in a high-yielding region. Yet, their statistical significances varied considerably, likely in part due to local conditions. Olk et al. (2021) reported the same findings. Modest agronomic responses can still be profitable economically if commodity prices are favorable, as the cost of many humic products is low. If this study were repeated on maize in a lower-yielding region or on another crop, a different array of results may well be found. More work is needed to determine the efficacy of humic products in promoting plant growth for the wide ranges of crop types, soil types, and environmental conditions that typify production agriculture.

## Conclusion

Application of the Yield Igniter^®^ humic product to maize production fields in the western US Maize Belt resulted in frequent positive responses by maize growth. Total leaf area increased significantly in all the eight field treatments where it was measured. Grain yield, as measured by combine for five site—year combinations, increased in all cases, and grain weight based on hand-sampled yield components for the on-farm survey increased in each of the 3 years. Increases were modest agronomically in this high-yielding region and varied in statistical significance, but the low cost of the humic product meant that it could provide profitable returns, depending on grain prices. Other yield components responded generally in positive manners, but as with the combine grain yield, their statistical significance varied and were often of modest magnitude. Even in this high-yielding region, the humic product demonstrated the capability to improve crop growth. Results could differ in other field studies depending on multiple factors, including humic product, crop type, crop management practices, and environmental conditions.

## Data Availability Statement

The original contributions presented in the study are included in the article/supplementary material, further inquiries can be directed to the corresponding author/s.

## Author Contributions

DD and DO contributed to the design of the studies and conducted or supervised all plant and soil samplings, oversaw the sample analyses, developed the interpretations, and drafted the manuscript. CC coordinated field management practices and harvesting of some of the replicated field trials in Central Iowa. All authors reviewed and approved the manuscript.

## Conflict of Interest

CC was employed by the company Ag Logic Distributors. The remaining authors declare that this study received funding from both USDA-ARS and the Ag Logic Distributors Company through USDA-ARS Trust Fund Cooperative Agreement 58-3625-9-563. Ag Logic Distributors Company agreed to the publication of this manuscript but was not involved in sample collection, analysis, interpretation of data, the writing of this article, or the decision to submit it for publication.

## Publisher’s Note

All claims expressed in this article are solely those of the authors and do not necessarily represent those of their affiliated organizations, or those of the publisher, the editors and the reviewers. Any product that may be evaluated in this article, or claim that may be made by its manufacturer, is not guaranteed or endorsed by the publisher.

## Footnotes

1 Reference to any specific commercial product is only for the information of the public and does not constitute endorsement or recommendation by the US government.

## Abbreviations

ANOVA: analysis of variance
LSD: least significant difference
V: leaf vegetative growth stage.
